# A comparative study of brine solutions as completion fluids for oil and gas fields

**DOI:** 10.1038/s41598-024-63303-5

**Published:** 2024-06-01

**Authors:** Parsa Kazemihokmabad, Ehsan Khamehchi, Javad Mahdavi Kalatehno, Reza Ebadi

**Affiliations:** Department of Petroleum Engineering, Amirkabir University of Technology, Tehran, Iran

**Keywords:** Completion fluid, Packer fluid, Formation damage, Chloride base fluid, Potassium acetate, Potassium formate, Chemistry, Energy science and technology, Engineering

## Abstract

Completion fluids play a vital role in well-related processes within the oil extraction industry. This article presents a comprehensive study of the properties and performance of various brine solutions as completion fluids for different well and reservoir conditions. Attributes examined include density, corrosion resistance, temperature stability, compatibility with formation fluids, clay swelling potential and influence on wettability. The research highlights the significance of selecting appropriate completion fluids to optimize well and reservoir operations. Zinc chloride emerges as an excellent option for high density applications, while sodium chloride and potassium formate solutions are ideal for extreme cold conditions. Potassium acetate outperforms calcium chloride and potassium chloride and has excellent pH stability. The compatibility of completion fluids with formation water has been observed to be excellent, with no sedimentation or emulsion formation. Potassium acetate also experiences minimal clay swelling, making it suitable for clay-rich formations. On the other hand, calcium chloride has a higher clay swelling than most of the brines tested, making it less suitable for sandstone formations with a higher clay content than these brines. The research evaluates the water-wetting abilities of completion fluids in carbonate and sandstone formations. Potassium chloride and zinc chloride have the most significant impact in carbonate formations, while potassium acetate and potassium formate excel in sandstone formations. This study provides a comprehensive understanding of completion fluids, facilitating informed decisions that maximize operational efficiency, protect reservoir integrity, and enhance hydrocarbon recovery. The appropriate selection of completion fluids should align with specific well and reservoir conditions, considering the priorities of the application.

## Introduction

In the realm of drilling operations, a well takes shape, starting from the surface and extending down to the reservoir. During this complicated building process, different parts are built at different times. For each part, special drilling fluids must be carefully chosen to match the rock structure and reduce any problems that might arise during the drilling. The choice of drilling fluids is pivotal and largely depends on the selected completion methods. Reservoir sections, in particular, are often navigated with the aid of a proprietary solution known as reservoir drilling fluid. This specialized system serves the dual purpose of minimizing formation damage in the vicinity of the well and optimizing the overall production capacity. Once the drilling phase reaches its culmination and additional equipment installation becomes imperative, a seamless transition takes place. The drilling fluid is replaced by a different kind of solution known as completion fluid. This switchover operation is a well-thought-out process, employing a series of specially designed spacers to cleanse the well, prepare the casing with water (if a non-aqueous drilling fluid was utilized for drilling the reservoir), and prevent any contamination during the shift from drilling fluid to completion fluid^1,2^. Completion fluid, as its name implies, is the linchpin in finalizing an oil or gas well. This unique fluid is introduced into the well to pave the way for the final operations before production kicks off. These operations include the installation of production liners, packers, downhole valves, and production zone perforation. Moreover, completion fluid acts as a safety net, allowing for well control in cases of downhole equipment failure without inflicting damage on the completion components or the formation. While the most common composition of completion fluids comprises brines, consisting of chlorides, bromides, formates, nitrates, and phosphates, theoretically, any fluid with the right density and flow characteristics can be employed^3^. At the conclusion of well completion, packer fluid lingers in the annular space situated between the casing and the production tubing, creating hydrostatic pressure. This particular formulation of completion fluid must possess key characteristics: it must be free of solids, possess resilience against changes in viscosity over extended periods, and be non-corrosive when in contact with the well and its components^1,4–6^. Designing a novel completion fluid commences with the meticulous selection of clear brines that align with the desired density. After that, the clear brines are carefully chosen by looking at a number of other factors, such as the true crystallization temperature, the pressure crystallization temperature, the ability to stop gas hydrate formation, the effect on metal corrosion, viscosity, temperature stability, pH balance, environmental concerns, cost implications, and the chance of damaging the formation^1,4^. Formation damage is a pivotal aspect in the realm of oil and gas production. It is a concept that significantly influences the industry, affecting various stakeholders and having a considerable impact on companies operating in the oil field^7^. Formation damage refers to the deterioration of petroleum formation permeability due to a variety of adverse processes. This unwelcome issue rears its head at different stages of oil and gas extraction, including drilling, production, hydraulic fracturing, and workover operations^8^. Formation damage manifests through four primary mechanisms: mechanical, chemical, biological, and thermal. Each of these can be further subdivided into specific sub-mechanisms.

Various salts are discussed in this article. Among the tested brine options, zinc chloride stands out. This salt has been employed in the oil industry for centuries. However, it poses environmental risks and can accumulate in the bodies of aquatic organisms. Additionally, it has a relatively short lifespan in terms of maintenance^9^. Another fluid under investigation is potassium formate which is a formate-based fluid. These fluids were initially developed in 1990 for high-temperature applications. Since 2000, they have gained widespread use in Canada, China, and Russia. Reports from these fields often highlight improved well stability, increased drilling speed, excellent thermal stability, and higher production rates after well completion^10^. Formate-based fluids encompass a wide range of densities. They do not cause formation damage and promote clay stability^11–13^. Formate brines are environmentally friendly and do not pose issues for personnel^14^. Common formate fluid salts include sodium formate with a density of 1.33 g/cm^3^, potassium formate with a density of 1.59 g/cm^3^, and cesium formate with a density of 2.3 g/cm^3^^15^. The first usage of potassium formate, along with manganese tetroxide, was in the Gulfaks field in Norway^13^.

In 1993, downs et al. conducted several experiments on formate brine based completion fluids and compared them with halide brine based completion fluid based on existing literature; But they did not perform any tests on halide brines and didn’t compare these two types of brines under the same experimental conditions. They also didn’t investigate the effect they have on wettability^11^. In 2002, Lomba et al., studied the rheological properties and temperature stability of formate-based fluids. The study evaluated the viscosity of three fluids: sodium formate, potassium formate, and potassium/cesium formate, concerning shear stress at different temperatures. They however, did not perform those same evaluations on the more commonly used halide brine; nor did they investigate the effect the fluids have on formation damage^16^. In 2003, Javora et al. examined the temperature stability and corrosion of formate fluids. Their findings revealed that utilizing certain buffers mitigated the effect of acid gases like H_2_S and CO_2_ on the corrosion caused by formate fluids. the decomposition and corrosion values of formate fluids were dependent on the steel that came into contact with them. However, they did not compare their results with halide-based completion fluids; or conduct any tests on the effect the fluids have on formation damage.^17^. Between 1999 and 2004, approximately 300 wells were drilled and completed in Alberta and British Columbia using formate fluids^18^. In 2013, Gomez et al., conducted experiments demonstrating that geological aspects can aid in comprehending clay swelling after contact with different fluids. This knowledge is vital for selecting the appropriate additives to prevent this phenomenon. Their research revealed that in clay formations with robust illite-rich layers, sodium chloride brine outperforms potassium chloride brine as a clay swelling inhibitor. Furthermore, they observed that calcium chloride brine suffices as a swelling inhibitor in silty shale formations with sandstone lenses^19^. In 2013 Chen et al., investigated the effect of propargyl alcohol on stress corrosion cracking of super 13Cr stainless steel in carbon dioxide saturated CaCl2 based completion fluid in the presence of 0.2 percent acetic acid; but no investigation was conducted on other types of completion fluid; nor were there any tests performed on the effects the additive has on formation damage. The results indicate that super 13Cr steel is susceptible to SCC without addition of propargyl alcohol inhibitor^20^. In 2015, Xuanpeng et al., developed a novel completion fluid by incorporating various additives into calcium chloride brine while not performing any tests on other types of brines; nor conducting any experiments on the novel fluid’s temperature stability or wettability change it causes. This fluid, used in four wells with a density range of 1.2–1.55 g/cm^3^ and exhibiting steel corrosion akin to fresh water, has a freezing point below – 10 °C. It includes additives like sediment inhibitors, corrosion inhibitors, clay swelling inhibitors, and weighting agents. Importantly, this designed fluid is compatible with formation water containing salts such as sodium bicarbonate, sodium sulfate, calcium chloride, and magnesium chloride, with no precipitation issues^21^. In 2016, Fleming et al., only looked into the formation damage that formate fluids cause but did no experiments on halide brines; nor did they conduct any experiments on their behaviour in extreme temperatures or the corrosion caused by them. Results from their core flood tests indicated that the return permeability affected by cesium/potassium formate fluid was estimated to be 76%, but this formation damage was resolved by the production well opening around the well^22^. In 2017, Al Moajil et al., conducted tests to evaluate the potential formation damage in gas wells caused by a sodium chloride-based completion fluid with a pH range of 10–10.5. He, however, conducted no experiments on other brine based completion fluids. they also performed no tests on the wettability change and corrosion caused by the completion fluids. Following the brine core flood test, they observed a permeability reduction of 12 to 31%. This decrease was primarily attributed to the fluid’s high pH and its incompatibility with formation water. Additionally, liquid precipitation occurred at 149 °C^23^. In 2018, Xu et al., conducted research on the corrosion of formate brines. they performed corrosion tests on formate fluids with different grades of steel, including N80, G3, and TP110SS, under laboratory conditions. In laboratory settings, formate fluids exhibited minimal corrosion. However, under conditions of high temperature and pressure, in the presence of CO_2_ and H_2_S gases, the corrosion caused by formate fluids significantly increased; so they designed a corrosion system with anti corrosion additives that overcomes this problem; but they didn’t investigate the effect this system has on formation damage and ignored other types of fluids^24^. In 2020, Tariq et al., introduced a completion fluid additive known as polyoxyethylene quaternary ammonium gemini surfactant, which effectively prevents clay swelling. They conducted experiments involving core flood sandstone cores with a high clay content and compared the results to potassium chloride and sodium chloride solutions. But they did not compare it to formate based solutions. They also didn’t conduct any experiments on the corrosion or the change in wettability caused by the completion fluids. The findings indicate that flooding with a sodium chloride solution led to an 80% reduction in permeability, while potassium chloride resulted in a 38% reduction. In contrast, the gemini surfactant solutions did not significantly decrease permeability^25^. In 2022, Avula et al., prepared three fluids based on potassium formate with varying weights and assessed their rheological properties at different temperatures. They discovered that because of its optimal characteristics, 60% w.l. potassium formate based fluid could be better suited for drilling, completion or workover applications. However, they conducted no experiments on the effect this concentration has on formation damage; they also ignored other types of fluids^26^. In 2022, Ahmed Khan et al., performed several core flood experiments on chloride based and ionic fluid based completion fluids by using Scioto sandstone core samples, but no experiments were conducted on organic based ones. There were also no experiments done on the corrosion or wettability change caused by the completion fluids. They concluded that unlike the chloride based completion fluids, the ionic based completion fluid caused almost no reduction in permeability^27^.

As can be seen from existing literature, although several studies have been conducted on common brines, none of these studies have been conducted on many brines under the same experimental conditions. Furthermore, the mechanisms of formation damage have either not been explored or have not been comprehensively investigated in prior research.To address this gap, a comparative analysis presented in Table 1 contrasts the documented findings with new empirical results, offering a detailed and systematic assessment of various brines and the corresponding mechanisms of formation damage. This paper endeavors to investigate the numerous necessary properties of completion fluids for various medium-weight and heavy brines under the same experimental conditions. the necessary properties also include evaluating the formation damage that these solutions may cause in different reservoir conditions. The work steps of the investigations are respectively density assessment, corrosion testing, viscosity analysis, stability testing at high and low temperatures, as well as assessments of compatibility with various formation waters, oil compatibility, clay swelling, and the wettability alteration of reservoirs by completion fluids.

**Table 1:** Literature review.

Author-year	Tested brines	Experiments	Conclusions
Downs et al. 1993	Formate based	HSE Corrosion Polymer compatibility Formation water compatibility Core flood	Non-hazardous and compatible with oilfield hardware Environmentally responsible and biodegradable Help to protect viscosifiers and fluid-loss polymers against thermal degradation
Lomba et al. 2002	Sodium/potassium/cesium formate based	Rheology	An analytical expression was devised to establish a correlation between shear stresses and temperature in general drilling fluids
Javora et al. 2003	Formate based fluids and buffers	Temperature stability Corrosion	Corrosion rates of mild steel, 13 chrome, and 22 chrome in formate brines are low under nitrogen environments Buffer additives mitigate, to some extent, the corrosive effects of acid gases on formate brines
Gomez et al. 2013	Potassium/sodium/calcium chloride—amine based	Clay swelling	In clay formations with robust illite-rich layers, sodium chloride brine outperforms potassium chloride brine as a clay swelling inhibitor Calcium chloride brine suffices as a swelling inhibitor in silty shale formations with sandstone lenses
Chen et al. 2013	Calcium chloride—propargyl alcohol—acetic acid	Corrosion	Super 13cr steel is susceptible to scc in CaCl_2_ based completion fluid in the presence of 0.2 percent acetic acid without addition of propargyl alcohol inhibitor
Xuanpeng et al. 2015	Calcium chloride—sediment inhibitors—corrosion inhibitors—clay swelling inhibitors—weighting agents	Corrosion Clay swelling Fluid compatibility	Developed a novel completion fluid by incorporating various additives into calcium chloride brine
Fleming et al. 2016	Potassium/Cesium Formate Based	Coreflood Cfd modeling Clay swelling	The coreflood study has shown a permissible return permeability of 76% when the valemon core was extended in contact with formate
Al moajil et al. 2017	Sodium chloride based—biocide—sodium sulfite—caustic soda—filming amine	Coreflood Temperature stability	They observed a permeability reduction of 12–31%. This decrease was primarily attributed to the fluid's high ph and its incompatibility with formation water Liquid precipitation occurred at 149 degrees celsius
Xu et al. 2018	Formate based—anti corrosion additives	Corrosion	Introduces an appropriate adjusted treatment, assesses the corrosion effect under different conditions, and develops a formate packer fluid system capable of meeting the user requirements under the conditions of high temperature, high pressure, high CO_2_ and high H_2_S content
Tariq et al. 2020	Potassium/sodium chloride—polyoxyethylene quaternary ammonium gemini surfactant	Coreflood	Introduced a completion fluid additive known as polyoxyethylene quaternary ammonium gemini surfactant, which effectively prevents clay swelling. Flooding with a sodium chloride solution led to an 80% reduction in permeability, while potassium chloride resulted in a 38% reduction
Avula et al. 2022	Potassium formate brine—additives	Rheological	Because of its optimal characteristics, 60% w.l. Potassium formate based fluid could be better suited for drilling, completion or workover applications
Ahmed khan et al. 2022	Chloride based completion fluids—ionic liqud	Coreflood	Unlike the chloride based completion fluids, the ionic liquid based completion fluid caused almost no reduction in permeability

## Materials

### Brine

In this study, brines of zinc chloride, calcium chloride, potassium chloride, sodium chloride, potassium acetate, and potassium formate, each with specific densities and pH levels, have been meticulously prepared. Table 2 below provides an overview of the characteristics of these brine solutions.

**Table 2:** Prepared brines and their characteristics.

Brines	Amount of salt (%wt)	Density (g/cm^3^)	pH	Turbidity (NTU)
CH_3_COOK	73	1.39	8–10.5	4.88
ZnCl_2_	81.2	1.89	0–2	17.4
CaCl_2_	42.85	1.42	6.5–8.5	11.3
HCOOK	70.9	1.52	6–8	20.7
KCl	25.4	1.17	6–7.5	5.1
NaCl	26.5	1.19	6–7.5	4.3

### Thin physical samples

**Figure 1:**
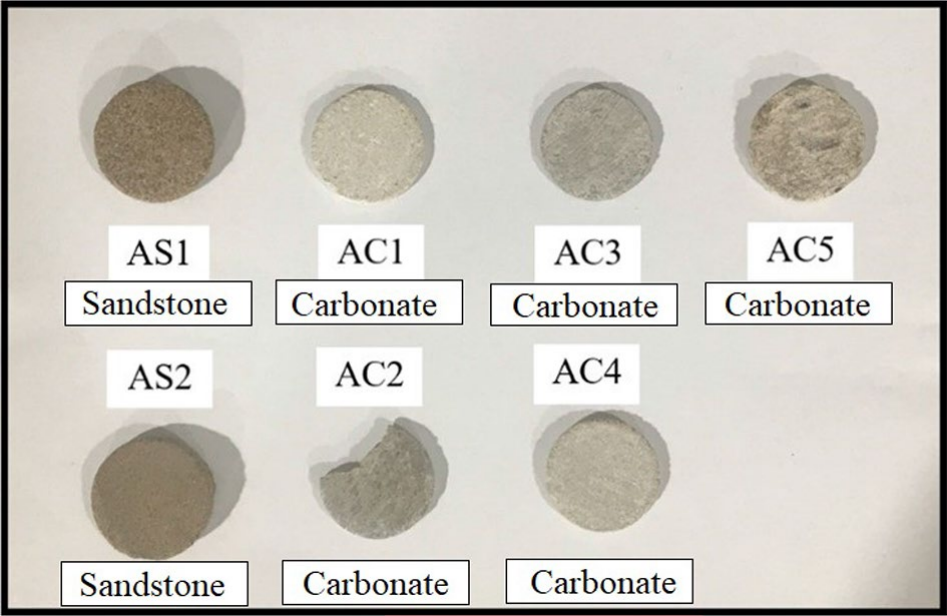
Thin physical samples.

As depicted in Fig. 1, thin physical samples of carbonate and sandstone, each measuring 0.08 inches in thickness, were meticulously prepared to assess the wettability alterations induced by the completion fluids.

### Corrosion coupons

Steel samples crafted from L80 alloys were employed for the corrosion evaluation. The characteristics of these steel samples are detailed in Table 3.

**Table 3:** Corrosion coupon characteristics.

Material	Length (mm)	Width (mm)	Thickness (mm)	Circle diameter (mm)
L80 coupon	58.78	20.14	4.16	6.06

### Formation fluid

In this study, formation water and crude oil were collected from an oil and gas field in southern Iran for experiments. These samples were gathered from various wells within the field and transported to the laboratory under controlled conditions. Subsequently, the samples underwent filtration, stabilization, and were stored in suitable containers for further analysis. The ionic constituents of the formation water and crude oil were determined using standard methods and instruments, including ion chromatography, gas chromatography, and mass spectrometry. The results are presented in Tables 4 and 5, respectively.

**Table 4:** Formation water compositions.

NH_4_ (mg/L)	SO_4_ (mg/L)	NO_3_ (mg/L)	NO_2_ (mg/L)	F (mg/L)	Br (mg/L)	Cl (mg/L)	PO_4_ (mg/L)
27.7	185	15	0.071	0.38	290	105.64	0.04

**Table 5:** Crude oil compositions.

Composition	CH_4_	C_2_H_6_	C_3_H_8_	i-C_4_H_10_	n-C_4_H_10_	i-C_5_H_12_	n-C_5_H_12_	C_6_H_14_ (mean)	C_7_H_16_ (plus)
Mole (%)	46.16	7.34	4.41	0.86	2.18	1.05	1.20	1.76	35.05
wt (%)	7.61	2.27	2.00	0.51	1.30	0.78	0.89	1.56	83.08

## Methods

### Density

Oil and gas reservoirs typically operate under high temperature and pressure conditions, necessitating the use of heavy-weight completion fluids to balance the fluid's hydrostatic pressure and the reservoir pressure. However, the required weight of the completion fluid varies based on the reservoir's depth. For shallow reservoirs with high pressure, a heavy and costly completion fluid (exceeding 100 pounds per cubic foot) may be essential. Conversely, for deeper reservoirs, medium-weight completion fluids with a lower cost might suffice, as the hydrostatic pressure on the reservoir increases with depth^4^. The density of a brine is determined by the type and concentration of the salt it contains. In this section, fluid density is measured using a pycnometer in accordance with ISO 13503-3-2006. This method entails weighing the pycnometer with the fluid, subtracting the weight of the empty pycnometer, and then dividing by the volume of the pycnometer^28^.

### Corrosion

In oil and gas well operations, the integrity of well structures is paramount. Corrosion tests are essential to evaluate the potential impact of completion fluids on well construction materials, such as L80 steel. The primary quality of a completion fluid is its non-corrosive nature, as brines, commonly used as base fluids, can induce corrosion, posing risks to wellbore pipes and equipment^24,29–32^. The corrosion testing procedures delineated in this section are conducted in strict adherence to the comprehensive guidelines specified in API RP13 standards. The testing sequence commences with the meticulous measurement of the L80 steel coupon's weight, employing a precision scale with four decimal places. Subsequently, the density and pH levels of the completion fluid are meticulously ascertained. Following these initial measurements, the completion fluid is combined with the coupon, and the entirety is positioned within a dedicated corrosion cell. This specialized cell subsequently undergoes placement within an oven set to an exacting temperature of 149 °C. Moreover, the cell is subjected to a pressure environment reaching 2.76 MPa, and the incubation period extends over a span of 72 h. Upon the conclusion of this exposure period, the cell is painstakingly opened, facilitating the careful separation of the completion fluid from the steel coupon. This sequence culminates with a comprehensive cleaning process of the coupon, followed by the recalibration of its weight. Ultimately, the extent of corrosion induced by the completion fluid is meticulously quantified, expressed in millimeters per year, utilizing the precise mathematical Eq. (1)^33^.

1 $$CR{ }\left( {mm/y} \right) = \frac{{weight{ }\;loss\left( {gr} \right) \times 3654.3152}}{{A\left( {cm^{2} } \right) \times metal{ }\;density{ }\;of{ }\;coupon\left( {\frac{g}{{cm^{3} }}} \right) \times time\;{ }of{ }\;exposure\left( {days} \right)}}.$$

### Temperature stability

Temperature stability is a critical aspect when evaluating the performance of completion fluids. Both low- and high-temperature stability tests are conducted to ensure that these fluids maintain their integrity under varying conditions.

### Low temperature stability

Low-temperature stability testing aims to determine the crystallization temperature of brine. This temperature marks the point at which solids begin to form. These solids can take the form of ice crystals (due to the freezing of water) or salt crystals precipitating because of reduced solubility. The precipitation of salt crystals not only reduces the solution’s density but can also obstruct pipelines, disrupt pumping operations, and clog filtration systems. Furthermore, it poses the risk of diminishing hydrostatic pressure in the brine, potentially leading to fluid loss and well control incidents. In this test, brine samples are carefully placed in a freezer at – 10 °C for a duration of 72 h. The test aims to observe the presence or absence of fluid sedimentation and crystallization.

### High temperature stability

For wells operating at significant depths, the completion fluids used may undergo precipitation or undergo alterations in their characteristics due to elevated temperatures. These temperature-induced changes can have a significant impact on the requisite attributes of the completion fluid. High-temperature stability tests are carried out to assess the clarity, density, and pH of the fluids under different temperature conditions, allowing for a comparative analysis. The test commences by transferring the selected brines into specially designed glass containers that are resistant to high temperatures and properly sealed to prevent liquid evaporation. The samples are subjected to a range of temperatures, including 25, 40, 60, 80, 100, and 120 °C. After a day at each temperature, measurements are taken to ascertain the density and pH of the samples, providing critical insights into the stability of completion fluids across a spectrum of operating conditions.

### Formation damage

Oil and gas wells can suffer from formation damage during any phase of drilling, completion, or stimulation. The formation can be harmed by the fluids that are used for these operations, as they may penetrate into it^8,34,35^. To avoid or minimize formation damage, it is essential to understand how the completion fluids affect the formation^36^. The completion fluid should have a pH between 7 and 9 because higher pH levels can weaken the sandstone reservoir’s structure^27^. This section examines the clay swelling caused by brines, the wettability changes induced by the fluid, and the compatibility of the brine with the formation fluids.

### Clay swelling

Clay swelling, a crucial consideration in oil and gas operations, is a phenomenon where water molecules envelop the crystalline structure of clay, causing an expansion in both distance and volume^37^. This expansion can detrimentally affect the permeability of the formation, potentially leading to decreased operational efficiency. In the context of drilling mud and water-based completion fluids, clay swelling is particularly problematic when these fluids infiltrate sedimentary rock formations. Such swelling can disrupt oilfield operations and result in substantial cost implications, making the mitigation of clay swelling a matter of paramount importance^38^. It is notable that clays with higher exchange capacities, such as smectite and their mixtures, exhibit pronounced swelling behavior, especially when situated within the larger pores of reservoir rock. This swelling creates nearly impermeable barriers to fluid flow. In contrast, clays with lower exchange capacities, such as kaolinite, illite, and chlorite, do not swell to the same extent upon hydration^39^. The addition of salt to drilling fluids is a common practice to minimize clay swelling since clay tends to swell more in pure water than in brine^40–42^.

The objective of this test is to evaluate the extent of clay swelling when exposed to various completion fluids. The procedure adheres to ASTM D5890 standards (ASTM 2006) for consistency and reliability^43^. Thus, the steps to perform the test are as follows:

1. Clay Preparation: Begin by isolating 2 g of bentonite clay powder.
2. Completion Fluid Primary: Place 90 cubic centimeters of the selected completion fluid into a 100 cubic centimeter test tube.
3. Incremental Clay Addition: Add 0.1 g of the 2 g of separated clay to the completion fluid at 10-min intervals until the clay is entirely hydrated.
4. Observation and Comparison: Following clay hydration, transfer a small amount of the completion fluid to an empty container containing the remaining 2 g of clay. Blend this mixture with the fluid from the test tube.
5. Volume Measurement: Return the mixture to the test tube, adding completion fluid as necessary to reach a total volume of 100 cubic centimeters.
6. Post-Hydration Observation: After a 24-h waiting period, observe and measure the volume of the clay, comparing it to its initial volume.

### Wettability alteration

Wettability, or a liquid's ability to stick to a solid surface, depends on a delicate balance between intermolecular interactions that stick (liquid-to-surface) and don’t stick (liquid-to-liquid)^44^. The ability of pore surfaces to hold water has a big effect on how different types of fluid move and settle in rock formations below the surface. This has a huge effect on how badly formations are damaged in oil reservoirs. Predicting how wettability will affect formation damage is hard because it changes over time and is affected by interactions between rocks and fluids as well as changes in the conditions of the reservoir^45,46^. Wettability, characterized by contact angles and correlated with surface tension, plays a pivotal role in oil reservoirs. Contact angles between 0° and 70° indicate highly water-wet rocks, while angles ranging from 110° to 180° signify a strong oil-wet nature. Intermediary wettability is attributed to contact angles between 70° and 110°^47^. This experiment aims to scrutinize the capacity of completion fluids to modify the wettability of reservoir formations, consequently enhancing production yields. The experimental procedure commences with the transformation of sandstone and carbonate- thin physical samples into oil-wet surfaces. To achieve this, these thin physical samples are immersed in a 0.01 M stearic acid solution and exposed to a temperature of 65.5 °C for 48 h. Following this treatment, the thin physical samples undergo a thorough wash with water and n-heptane. Subsequently, they are delicately positioned on the surface of water, and a droplet of kerosene, administered via a syringe, is dispensed beneath them. The interface of this droplet is meticulously captured through imaging with the assistance of Digimizer software, allowing for precise measurement of the contact angle. In instances where the thin physical samples exhibit an oil-wet disposition, they are introduced to the designated completion fluid and subjected to an aging similar to the previous step. This process is followed by the repetition of the aforementioned steps to ascertain whether these sections have undergone a transformation towards being water-wet or not^4,48,49^. This experiment tells us a lot about how completion fluids might be able to change how wet a reservoir is, which could lead to more oil being produced from hydrocarbon reservoirs.

### Fluid compatibility

In the context of this basin, the notion of fluid compatibility bears substantial importance. Fluid incompatibility surfaces when two fluids, upon their amalgamation, exhibit a tendency to separate into distinct phases or form an emulsion in the presence of salts or other solid constituents. Such incompatibility poses a notable risk of sediment formation within the porous media of the reservoir formation, potentially leading to pore blockage and detrimental formation damage. One of the most renowned instances of fluid incompatibility arises from the interaction of seawater with formation water. The characteristics that define this incompatibility hinge on the stark contrast between their respective ion compositions. Seawater is characterized by a high concentration of sulfate ions coupled with low levels of calcium, barium, and strontium ions. In stark contrast, formation water exhibits the inverse, boasting low sulfate ion concentrations and high levels of calcium, barium, and strontium ions. The outcome of their convergence is the precipitation of compounds such as calcium sulfate, barium sulfate, and strontium sulfate salts, which manifest as undesirable sediments^4,45,50,51^.

This section of our study revolves around a comprehensive assessment designed to probe the compatibility dynamics between completion fluids and various formation fluids. Notably, the latter encompasses formation water and crude oil. To scrutinize this intricate interplay, we have devised a series of meticulous experiments. Our aim is twofold: to ascertain the propensity for sediment formation or its absence after the amalgamation of desired completion fluids and diverse formation fluids across varying volume ratios, all subjected to elevated temperatures. The experimental protocol is orchestrated as follows:

1. Preparation of Fluid Mixtures: Completion fluids are meticulously blended with formation fluids in three distinct ratios: 1:1, 1:3, and 3:1.
2. Sealing in Resilient Glass Containers: These precisely concocted fluid mixtures are then carefully transferred to specialized glass bottles, engineered to withstand the rigors of autoclave temperatures.
3. Exposure to Elevated Temperatures: The sealed glass bottles, each representative of a distinct fluid mixture, are entrusted to an environment where temperature soars to 80 °C. This arduous environment serves as the crucible for assessments, simulating reservoir conditions in a controlled laboratory setting.
4. Post-Exposure Assessment: Following an aging period of 72 h, the samples are delicately extracted from their high-temperature crucible. Diligent examinations are directed towards the identification of sedimentation or the conspicuous absence thereof.

These well-planned experiments give a solid basis for figuring out how well different formation fluids and completion fluids work together. This helps in getting a full picture of the problems and interactions that might happen. Looking at how sediment forms when volume ratios change and temperatures rise helps in understanding how fluid compatibility affects the integrity and performance of reservoirs, which in turn helps in making smart decisions in real-world situations.

## Results and discussion

### Density

**Figure 2:**
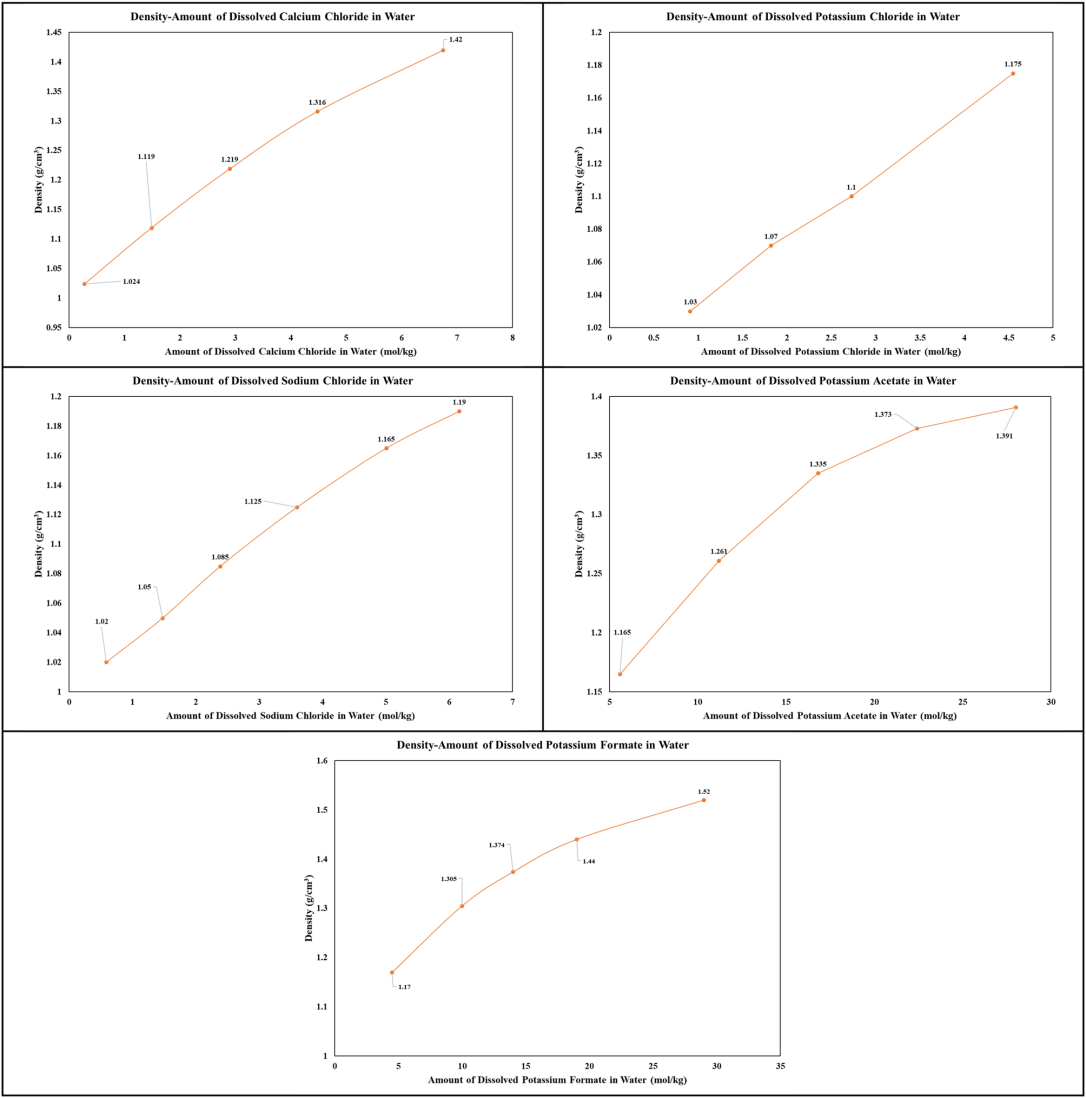
Graphs of the concentration of salt dissolved in water for brines.

The density of brine solutions plays a crucial role in their performance and suitability for a range of oil and gas operations. In this part, the density of different brine solutions was studied at different concentration levels. These solutions included calcium chloride, potassium chloride, sodium chloride, potassium formate, potassium acetate, and zinc chloride. The outcomes are visually depicted in Fig. 2, which effectively illustrates the connection between density and salt concentration for each respective brine solution.

An instructive insight gained from Fig. 2 is the pronounced density of calcium chloride brine relative to all chloride-based brines, with potassium chloride brine trailing in second place. This alignment with established literature reinforces the prevailing use of calcium chloride and potassium chloride as primary medium-density brines in activities related to well completion and workover operations. Moreover, the data reveals that, at a temperature of 25 ℃, potassium chloride brine achieves a higher peak density compared to its sodium potassium chloride counterpart. This finding implies a greater efficacy of potassium chloride in augmenting the density of water-based fluids when compared to sodium chloride.

Equally noteworthy, the data in Fig. 2 unveils an intriguing non-linear relationship between density and salt content for potassium formate and potassium acetate brines, setting them apart from their counterparts, which exhibit a linear behavior. This suggests the ease with which the density of these brines can be adjusted by modifying the concentration of potassium formate and potassium acetate. Furthermore, these brines exhibit moderate densities in contrast to their peers, rendering them particularly suitable for deployment in the context of deep, high-pressure wells. This is especially beneficial as the use of high-density fluids may give rise to formation damage or operational complications in such conditions. However, it is essential to acknowledge the challenges encountered during the course of this study, particularly the procurement of pure zinc chloride salt. The zinc chloride salt utilized in this study contained a noteworthy quantity of impurities, posing an impediment to generating a clear density graph. Despite this challenge, a solution was achieved by dissolving 760 g of zinc chloride in 300 cc of water. The resultant solution initially appeared cloudy but was successfully clarified following a filtration process. This observation indicates the substantial potential of zinc chloride as a high-density brine, a point of considerable significance.

### Corrosion

**Figure 3:**
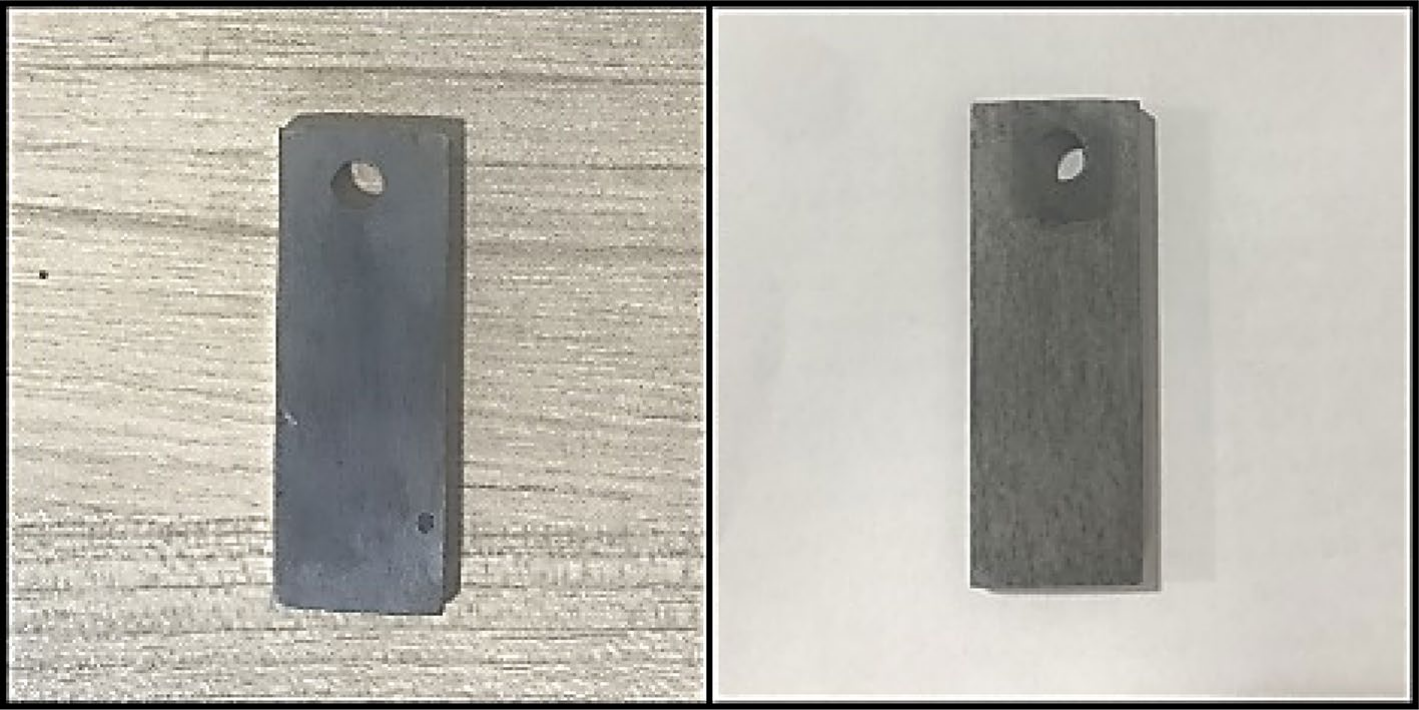
Sample of corrosion coupons after and before corrosion test.

In the context of corrosion testing, a series of comprehensive experiments were conducted at an elevated temperature of 149 °C, focusing on a range of brine solutions. The detailed results of these corrosion tests are meticulously documented in Table 6, providing a comprehensive overview of the corrosion characteristics exhibited by these fluids. Additionally, Fig. 3 serves as a visual representation of the transformation in corrosion coupons before and after these rigorous corrosion tests.

**Table 6:** The evaluation of fluid corrosion.

Brines	pH	Temperature (℃)	CR (mm/y)
CH_3_COOK	9.5	149	1.706
CaCl_2_	8.4	149	2.540
ZnCl_2_	2	149	0.076
HCOOK	7	149	0.046

A thorough analysis of Table 6 unequivocally reveals that both calcium chloride and potassium acetate exhibit higher corrosion rates compared to the other examined fluids. Intriguingly, calcium chloride demonstrates a notably higher corrosion rate than potassium acetate. In scenarios where corrosion rate is of paramount concern within a well, it becomes apparent that potassium acetate solution presents a more favorable option when contrasted with calcium chloride. In cases where these specific fluids are deemed indispensable for operational requirements, the judicious deployment of corresponding corrosion inhibitors is highly recommended. It is vital to underscore the significance of maintaining the pH of the completion fluid within the optimal range of 7–9.5. This pH range not only plays a pivotal role in corrosion control but also serves as a preventive measure against clay swelling. As elucidated in Table 6, the pH levels of all fluids, except for zinc chloride, steadfastly fall within the recommended pH range of 7–9.5. This observation is particularly remarkable given the fact that zinc chloride, despite its exceptionally low pH and the presence of zinc ions, typically associated with heightened corrosion rates, displays the lowest corrosion rate when juxtaposed with the other tested solutions. It is imperative to acknowledge that potassium chloride and sodium chloride solutions were not subjected to these specific corrosion tests. This decision was based on the extensive body of prior research conducted on these solutions and their distinctly low densities, rendering them unsuitable for inclusion in the current study.

### Temperature stability

#### Low temperature stability

**Figure 4:**
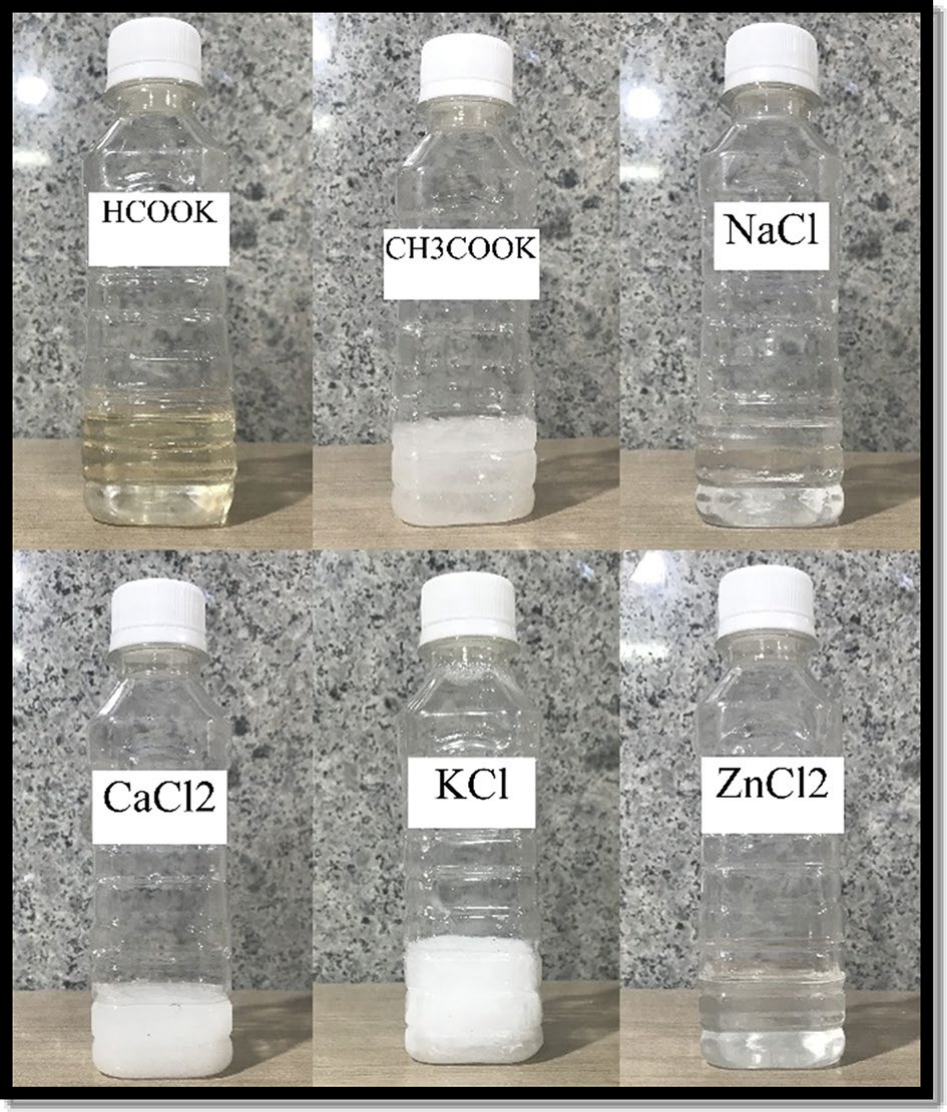
Brine solutions after low temperature stability test.

In the evaluation of low-temperature stability, a noteworthy distinction emerged among the tested brines. As depicted in Fig. 4, it’s evident that potassium acetate, potassium chloride, and calcium chloride brines experienced crystallization at a frigid temperature of – 10 °C. This crystallization phenomenon is a crucial consideration as it can potentially lead to blockages in pipelines, interference with pumping operations, and clogging of filtration systems. However, intriguingly, the potassium acetate solution exhibited notably less crystallinity compared to calcium chloride. On the other hand, zinc chloride, sodium chloride, and potassium formate brines demonstrated resilience, showing no discernible signs of precipitation or crystallization under these extreme cold conditions.

These findings underscore the importance of selecting the appropriate brine solutions for specific well conditions, particularly in regions where sub-zero temperatures are encountered. Because potassium acetate is less crystallized than calcium chloride, it is a better choice in these conditions because it is less likely to precipitate and get clogged when temperatures are low. This means that it works more reliably. Additionally, the exceptional stability that zinc chloride, sodium chloride, and potassium formate exhibit, further emphasize their suitability for use in wells where low-temperature stability is a crucial consideration. Their ability to remain free from crystallization at − 10 °C underlines their potential as reliable choices in oil and gas operations that confront extreme cold conditions.

#### High temperature stability

**Figure 5:**
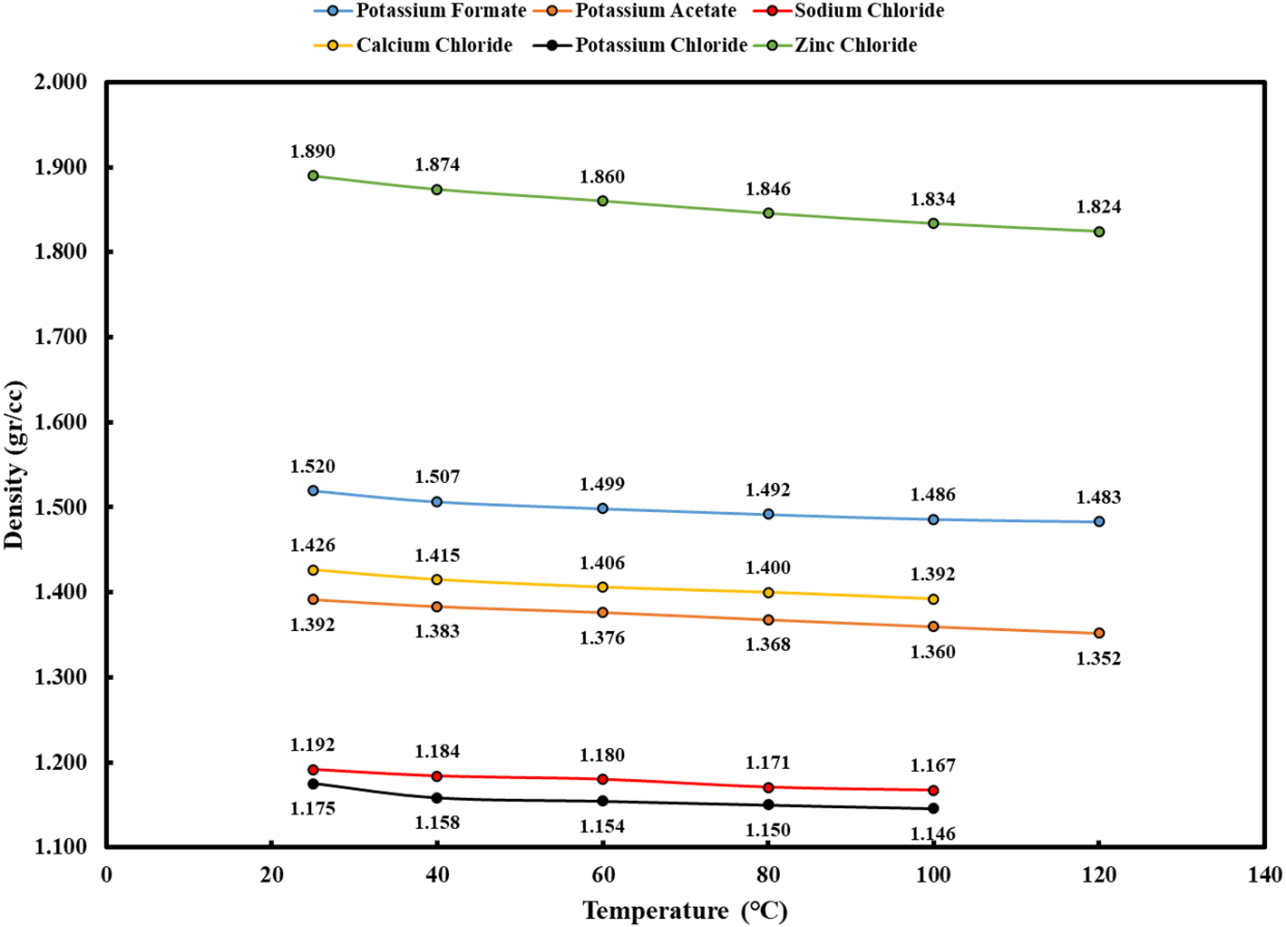
Density changes of brines with increasing temperature (after high temperature stability test).

**Figure 6:**
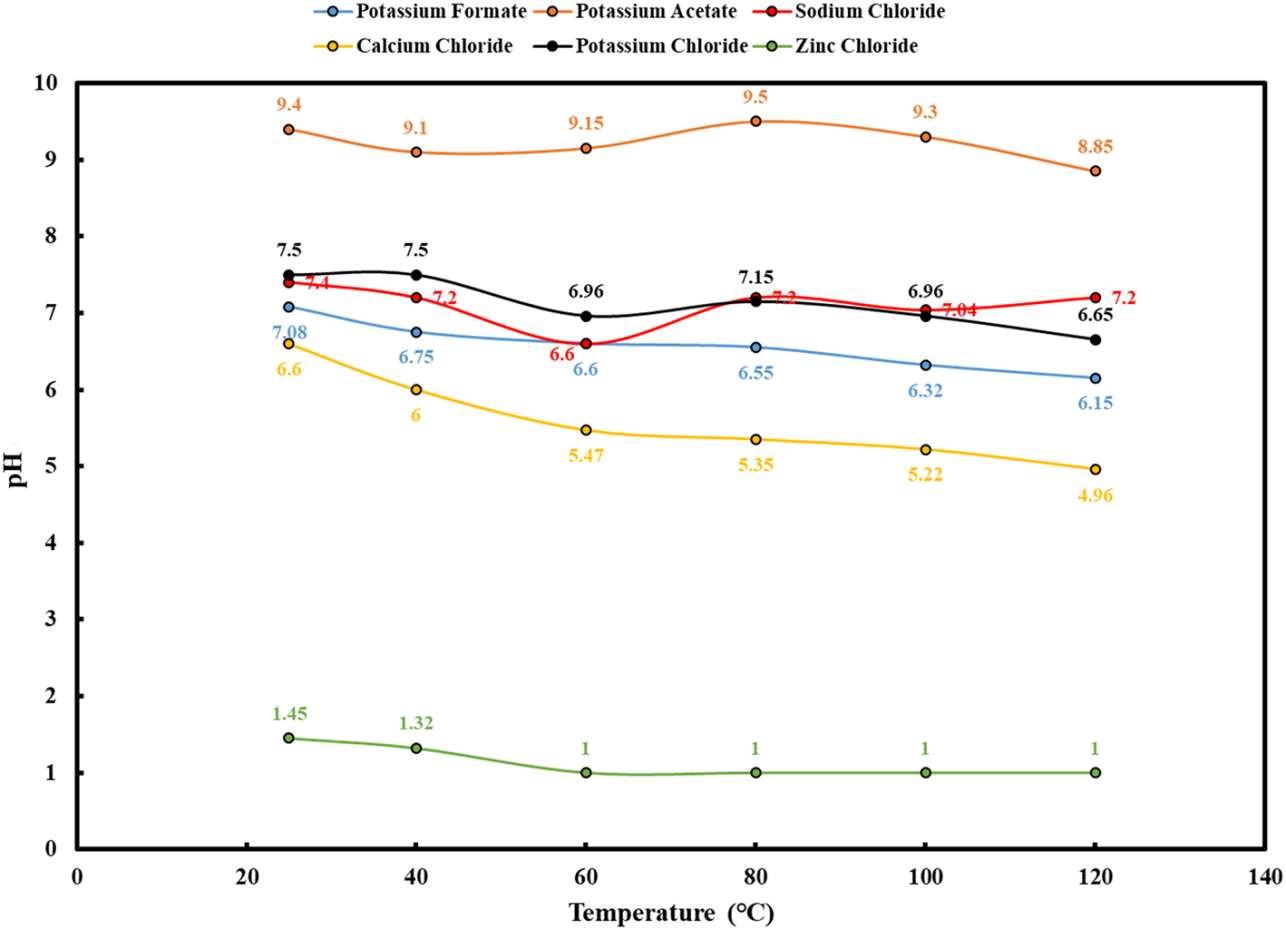
pH changes of brines with increasing temperature (after high temperature stability test).

**Figure 7:**
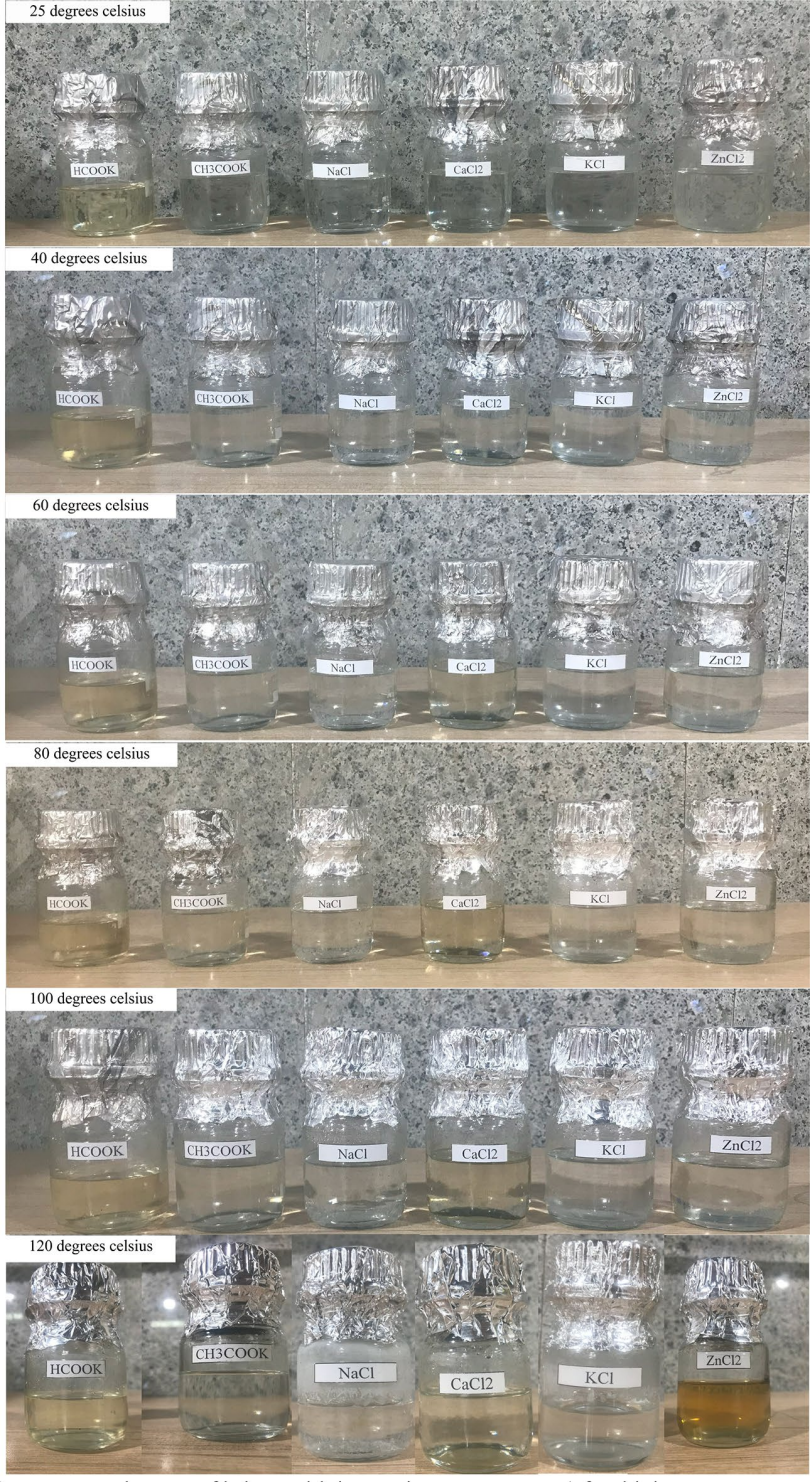
Appearance changes of brines with increasing temperature (after high temperature stability test).

High-temperature stability is a crucial aspect when assessing completion fluids, especially for wells exposed to elevated downhole temperatures. Figures 5 and 6 present the key findings regarding density and pH changes following high-temperature stability tests. Moreover, Fig. 7 provides visual insights into the outcomes of these experiments for each solution.

In the case of potassium formate brine, the test outcomes are illuminating. The increase in temperature from 25 to 120 °C resulted in a substantial drop in fluid density, reaching as low as 0.037 g per cubic centimeter. The pH also exhibited a noticeable decline within this temperature range, settling around 1. The results highlight the sensitivity of potassium formate brine to high-temperature conditions, with a significant impact on both density and pH. The high-temperature stability tests for calcium chloride brine also showed that its density and pH dropped. It dropped by about 0.034 g per cubic centimeter and had pH shifts that were not good when it was exposed to temperatures between 25 and 100 °C. However, the test at 120 °C faced challenges due to the extreme conditions, resulting in vaporization and unrealistic density values. Nonetheless, Fig. 7 illustrates that the solution remained completely clear despite these harsh conditions. As the temperature increased from 25 to 100 °C, potassium chloride brine showed a decrease in density of less than 0.029 g per cubic centimeter and a pH reduction of roughly 1 pH unit. Significant fluid vaporization hindered the test at 120 °C, as seen in the other brines, making density readings unrealistic. Nevertheless, Fig. 7 demonstrates the brine's ability to maintain clarity under these demanding conditions. Sodium chloride brine exhibited the lowest density reduction, approximately 0.025 g per cubic centimeter, as temperatures elevated from 25 to 100 °C. The pH experienced a moderate reduction of 0.4 pH units, with an initial gradual decrease followed by an increase within the 60–120-degree range. Despite facing challenges during the test at 120 °C due to vaporization, Fig. 7 displays the continued clarity of the solution. Contrastingly, the high-temperature stability test for zinc chloride solution unveiled more significant shifts. As the temperature escalated from 25 to 120 °C, the density decreased by approximately 0.066 g per cubic centimeter. Notably, the solution exhibited a color change to bright yellow, signifying the potential issues when employing zinc chloride solution in high-temperature wells. Careful consideration is advised when using this solution under such conditions. The temperature stability assessment of potassium acetate brine revealed unique characteristics. The density decreased to about 0.039 g per cubic centimeter as the temperature climbed from 25 to 120 °C. However, the pH displayed intriguing behavior. It decreased up to 60 °C and, contrary to expectations, increased until reaching 80 °C. Subsequently, as the temperature peaked at 120 °C, the pH decreased to 8.85. Figure 7 visually captures the slight yellowing of the solution as the temperature gradually increased. These observations suggest that potassium acetate solution exhibits higher density loss compared to other solutions, necessitating careful handling in high-temperature well applications.

### Formation damage

#### Clay swelling

**Figure 8:**
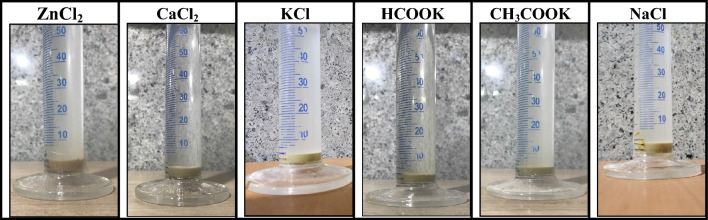
Clay swelling test of brines after 24 h.

The clay swelling tests were performed on five distinct fluids: zinc chloride, calcium chloride, potassium chloride, potassium acetate, and potassium formate. The outcomes of this investigation are summarized in Table 7, and a visual representation of the clay swelling test for these fluids is provided in Fig. 8.

**Table 7:** The clay swelling index of brines.

Brines	Swelling index (2g/cc)
ZnCl_2_	≈ 4
CaCl_2_	≈ 4
KCl	≤ 1
CH_3_COOK	≈ 3
HCOOK	≤ 4
NaCl	≤ 4

As depicted in Fig. 8, the clay immersed in the potassium chloride brine exhibited minimal swelling after a 24-h period. This result aligns with expectations, as potassium chloride brine contains potassium ions, which function as inhibitors of clay swelling, a well-established practice in the drilling and completion industry^38,52^.

Furthermore, upon closer examination, the swelling index for potassium formate brine is approximately 4 cubic centimeters per 2 g of clay. Surprisingly, it was observed that this fluid induced less swelling in comparison to calcium chloride. Figure 8 also reveals the behavior of the clay when subjected to potassium acetate. The presence of potassium ions in this salt mitigated significant swelling, resulting in a swelling index of approximately 3 cubic centimeters per two grams of clay. This data strongly suggests that potassium acetate brine is effective at reducing clay swelling, outperforming other brines except for potassium chloride. In the case of zinc chloride, Fig. 8 illustrates that this brine, similar to calcium chloride, exhibits a swelling index of 4 cubic centimeters per two grams of clay. This indicates that zinc chloride does not offer a substantial advantage over calcium chloride in mitigating clay swelling.

### Wettability alteration

The contact angles of different brines on carbonate and sandstone thin physical samples before and after aging (which was explained in the “Methods” section) were measured to investigate the effects of completion fluids on wettability. Aging simulated the long-term exposure of the rock to the completion fluid under reservoir conditions. five types of brines with varying salt concentrations and compositions were used: HCOOK (potassium formate), CH_3_OOK (potassium acetate), KCl (potassium chloride), CaCl_2_ (calcium chloride) and ZnCl_2_ (zinc chloride). Kerosene was used as the oil phase to create a two-phase system. Seven thin physical samples from carbonate and sandstone cores, labeled as AC1 to AC5 and AS1 to AS2, respectively, were prepared. Each thin physical sample was conditioned by applying a drop of kerosene to its surface and spreading it evenly.

**Figure 9:**
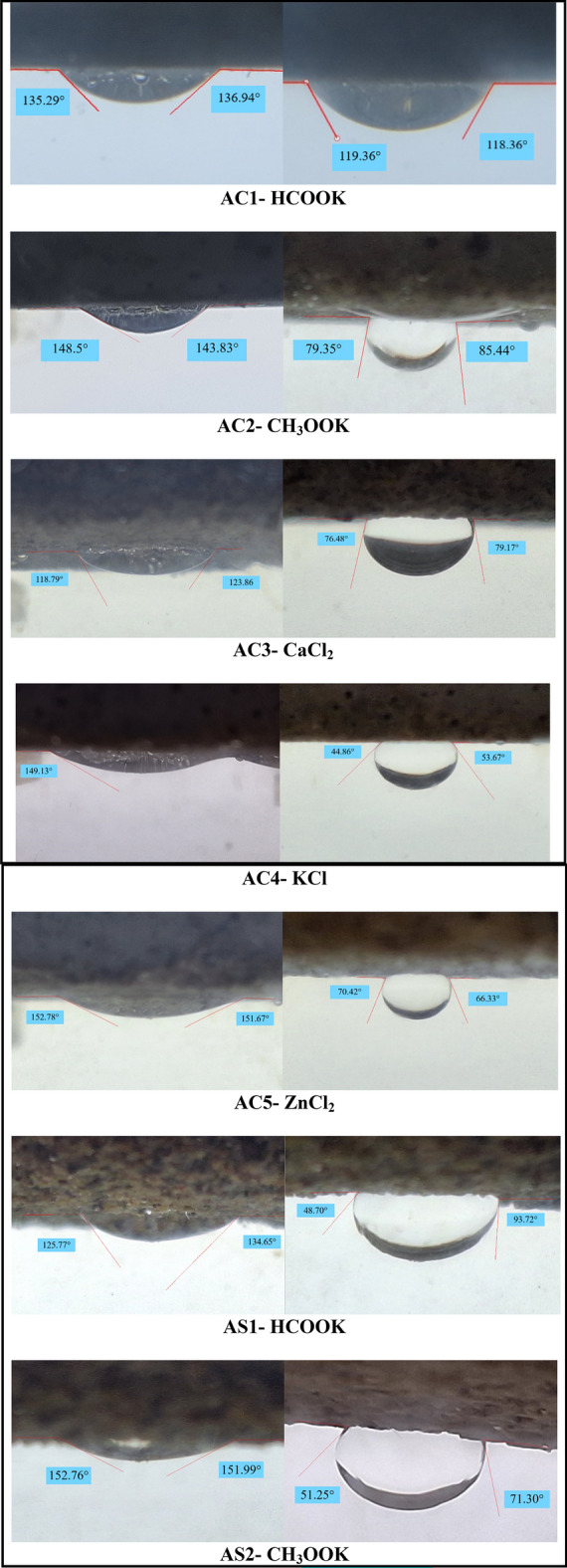
Wettability test of thin physical samples.

Figure 9 shows the images of thin physical samples sections before and after aging with different brines, along with the measured contact angles. Table 8 summarizes the results of the contact angle measurements and the corresponding wettability status of each thin physical sample after aging. The wettability status was classified into four categories based on the contact angle values: strongly water-wet (< 70°), intermediate wettability (70°–110°), and strongly oil-wet (> 110°).

**Table 8:** Wettability evaluation of thin physical samples.

Thin section	Brine	Average angle before aging	Average angle after aging	Angle difference	Wettability after aging
AS1	HCOOK	130.21	71.21	59	Intermediate water-wet
AS2	CH_3_OOK	152.375	61.275	91.1	Intermediate water-wet
AC1	HCOOK	136.115	118.86	17.255	Strongly oil-wet
AC2	CH_3_OOK	146.165	82.395	63.77	Intermediate water-wet
AC3	CaCl_2_	121.33	77.825	43.505	Intermediate water-wet
AC4	KCl	149.13	49.265	99.865	Intermediate water-wet
AC5	ZnCl_2_	152.225	68.375	83.85	Intermediate water-wet

All thin physical samples were initially oil-wet before aging, as indicated by their high contact angles with kerosene. This is consistent with the common observation that carbonate rocks tend to be oil-wet or mixed-wet due to the presence of organic matter and surface-active compounds. Sandstone rocks can also exhibit oil-wet behavior depending on the mineralogy, clay content, and pore structure. Different laboratory studies have shown that, by manipulating the brine composition and reducing salt content, carbonate rock can change its wettability to a lower oil-wet state. They suggest that the driving force behind the low alkalinity effect in carbonates is likely to be surface charge modification. The sensitivity of carbonate surface charge to brine salinity, pH value, and potential ions determining brine acidity has already been established in a number of studies. But The brine chemistry has been found to play a more important role than the salt content itself in said studies^53^. The results of this study show that different brines have similar effects on the wettability of carbonate and sandstone thin physical samples. Most brines cause significant changes in wettability and are placed in the strongly water wet and intermediate wettability category. This can be attributed to the ion exchange and surface complexation mechanisms that occur between the brine components and the rock surface. The increased concentration of cations in the brine can enhance the water-wetness of the rock by displacing the adsorbed organic matter and increasing the negative charge density on the surface. In the other case, the changes are different. AC1 is when the wettability of HCOOK does not change much from oil-wet to water-wet and is placed in the strongly oil-wet category. This may be because HCOOK solution has a low pH and a high salinity, which can preserve or enhance the oil-wetness of the carbonate rock by reducing the ion exchange and surface complexation effects; or because formate brines may have an adverse effect on wettability alteration. These revelations underscore the paramount importance of selecting the appropriate completion fluid for the prevailing reservoir rock. It has the potential to exert a profound influence on wettability, subsequently impacting oil recovery efficiency. By opting for a completion fluid that can recalibrate the rock’s wettability from oil-wet to water-wet, capillary forces restraining oil in the pores can be mitigated, and relative oil phase permeability can be enhanced. Conversely, choosing a completion fluid that leaves the reservoir rock’s wettability unaffected or exacerbates oil-wet tendencies may hamper oil production by amplifying residual oil saturation and diminishing relative oil phase permeability.

### Fluid compatibility

**Figure 10:**
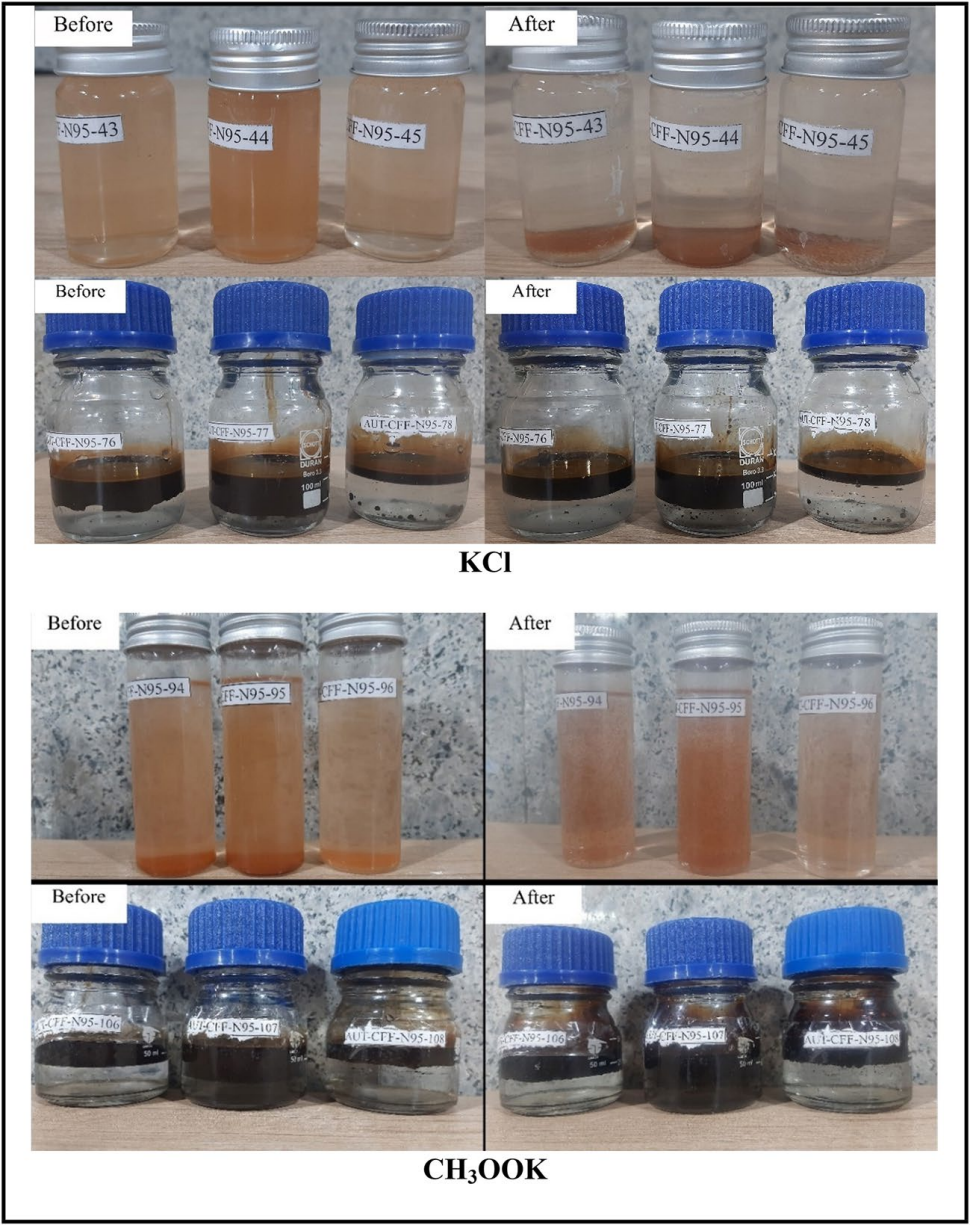

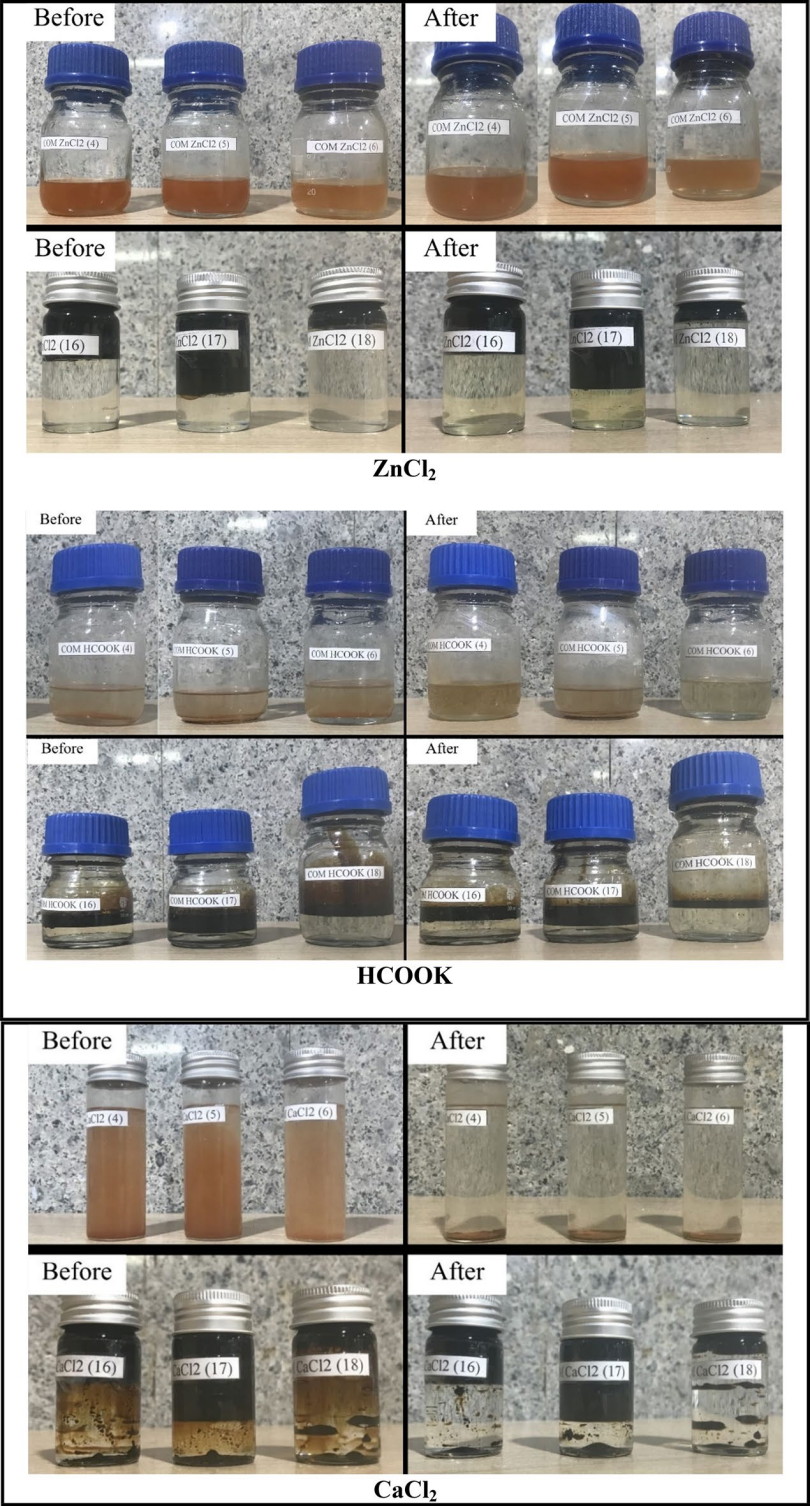
Before and after compatibility test samples solution.

This critical phase of our study delves into the intricate dance of compatibility between our carefully selected completion brines, formation water, and crude oil. The acquired results, unveiled within Table 9, find visual representation in Fig. 10, offering a comprehensive understanding of this complex interplay.

**Table 9:** Formation fluid compatibility test.

Code	Brine	Formation fluid	Brine ratio	Formation fluid ratio
AUT-CFF-N95-43	KCl	Formation water	1	1
AUT-CFF-N95-44	KCl	Formation water	1	3
AUT-CFF-N95-45	KCl	Formation water	3	1
AUT-CFF-N95-76	KCl	Crude oil	1	1
AUT-CFF-N95-77	KCl	Crude oil	1	3
AUT-CFF-N95-78	KCl	Crude oil	3	1
AUT-CFF-N95-94	CH_3_OOK	Formation water	1	1
AUT-CFF-N95-95	CH_3_OOK	Formation water	1	3
AUT-CFF-N95-96	CH_3_OOK	Formation water	3	1
AUT-CFF-N95-106	CH_3_OOK	Crude oil	1	1
AUT-CFF-N95-107	CH_3_OOK	Crude oil	1	3
AUT-CFF-N95-108	CH_3_OOK	Crude oil	3	1
COM ZnCl_2_ (4)	ZnCl_2_	Formation water	1	1
COM ZnCl_2_ (5)	ZnCl_2_	Formation water	1	3
COM ZnCl_2_ (6)	ZnCl_2_	Formation water	3	1
COM ZnCl_2_ (16)	ZnCl_2_	Crude oil	1	1
COM ZnCl_2_ (17)	ZnCl_2_	Crude oil	1	3
COM ZnCl_2_ (18)	ZnCl_2_	Crude oil	3	1
COM HCOOK (4)	HCOOK	Formation water	1	1
COM HCOOK (5)	HCOOK	Formation water	1	3
COM HCOOK (6)	HCOOK	Formation water	3	1
COM HCOOK (16)	HCOOK	Crude oil	1	1
COM HCOOK (17)	HCOOK	Crude oil	1	3
COM HCOOK (18)	HCOOK	Crude oil	3	1
COM CaCl_2_ (4)	CaCl_2_	Formation water	1	1
COM CaCl_2_ (5)	CaCl_2_	Formation water	1	3
COM CaCl_2_ (6)	CaCl_2_	Formation water	3	1
COM CaCl_2_ (16)	CaCl_2_	Crude oil	1	1
COM CaCl_2_ (17)	CaCl_2_	Crude oil	1	3
COM CaCl_2_ (18)	CaCl_2_	Crude oil	3	1

The results show that all completion fluids are compatible with formation water, as no sediment formation was observed in any of the mixtures. This indicates that the completion fluids do not react with the formation water or cause any precipitation or scaling. This is desirable for maintaining the porosity and permeability of the reservoir rock. The results also show that all completion fluids are compatible with crude oil, as no sediment formation was observed in any of the mixtures. This indicates that the completion fluids do not interact with the crude oil or cause any emulsion or asphaltene deposition. This is beneficial for enhancing oil mobility and recovery. The only exception was sodium chloride solution, which was excluded from the investigation due to its very low density. Sodium chloride solution may not be suitable for this reservoir, as it may cause density-driven convection and fingering phenomena that can reduce the sweep efficiency and oil recovery. These findings demonstrate the potential suitability of these completion fluids for real-world reservoir applications. The compatibility of these brines with formation water and crude oil implies that they can be used safely and effectively without causing any adverse effects on reservoir performance.

## Conclusion

In conclusion, the comprehensive testing of various brine solutions for their efficacy as completion fluids has revealed a range of unique properties and characteristics. The results of these experiments shed light on the suitability of these fluids for different well and reservoir conditions. The findings can be summarized as follows:

- Zinc chloride stands out with the highest maximum density of 1.89 g per cubic centimeter, making it a notable option for applications requiring high-weight completion fluids. Potassium acetate and potassium formate with maximum densities of 1.39 and 1.6 g per cubic centimeter exhibit a gradual decrease in density with increasing salt concentrations.
- Zinc chloride, sodium chloride, and potassium formate solutions demonstrate exceptional resistance to precipitation or crystal formation when subjected to extreme cold conditions of – 10 °C, making them ideal choices for offshore operations. Potassium acetate also outperforms calcium chloride and potassium chloride.
- Temperature sensitivity is a consideration, with sodium chloride displaying the least density reduction (0.025 g per cubic centimeter) and potassium acetate exhibiting the most significant decrease (0.04 g per cubic centimeter) after zinc chloride (0.066 g per cubic centimeter). Zinc chloride should be used cautiously in high-temperature wells. pH level reductions vary, with potassium acetate having the highest pH and calcium chloride the lowest after zinc chloride. Potassium acetate also shows superior pH stability compared to potassium formate.
- The compatibility of these fluids with formation water is excellent, with no sediment or emulsion formation with crude oil observed.
- Bentonite clay swelling is minimal in potassium acetate brine (≈ 3.2 g/cc), second only to potassium chloride (≤ 1.2g/cc). Calcium chloride, on the other hand, exhibits the highest clay swelling (≈ 4.2 g/cc), making it unsuitable for formations with a high clay content.
- The ability of completion fluids to water-wet carbonate and sandstone formations varies. Potassium chloride and zinc chloride show the most significant impact on water-wetting carbonate formations by reducing the contact angle by 99.865° and 83.85° respectively, while potassium formate has a minimal effect (17.255°).
- Despite their high pH, calcium chloride solution cause the highest corrosion (2.540) amongst the fluids.

In summary, the choice of the right completion fluid depends on the specific well and reservoir conditions at the time, considering the priority characteristics for the given application. Each solution has its own unique advantages and disadvantages, and this research provides valuable insights to guide such selection processes. Ultimately, the optimal completion fluid should be chosen to best meet the needs of the specific situation and objectives.

## Data Availability

All data generated or analyzed during this study are included in this published article.
